# Aldehyde Dehydrogenase 2 Ameliorates Chronic Alcohol Consumption-Induced Atrial Fibrillation through Detoxification of 4-HNE

**DOI:** 10.3390/ijms21186678

**Published:** 2020-09-12

**Authors:** Lung-An Hsu, Feng-Chun Tsai, Yung-Hsin Yeh, Chi-Jen Chang, Chi-Tai Kuo, Wei-Jan Chen, Hsin-Yi Tsai, Gwo-Jyh Chang

**Affiliations:** 1 Cardiovascular Division, Department of Internal Medicine, Chang Gung Memorial Hospital, Chang Gung University College of Medicine, No. 5, Fu-Shin Road, Kwei-Shan, Taoyuan 33305, Taiwan; yys0tw@yahoo.ca (Y.-H.Y.); chijenformosa@gmail.com (C.-J.C.); chitai@adm.cgmh.org.tw (C.-T.K.); wjchen@adm.cgmh.org.tw (W.-J.C.); sunny66house@yahoo.com.tw (H.-Y.T.); 2 Division of Cardiac Surgery, Chang Gung Memorial Hospital, Chang Gung University College of Medicine, Tao-Yuan 33305, Taiwan; lutony@cgmh.org.tw; 3 Graduate Institute of Clinical Medical Sciences, Chang Gung University, Tao-Yuan 33305, Taiwan; gjchang@mail.cgu.edu.tw

**Keywords:** atrial fibrillation, aldehyde dehydrogenase 2, polymorphism

## Abstract

Aldehyde dehydrogenase 2 (ALDH2) is an enzyme that detoxifies reactive oxygen species (ROS)-generated aldehyde adducts such as 4-hydroxy-trans-2-nonenal (4-HNE). Previous meta-analyses have shown an increase in the risk of atrial fibrillation (AF) in patients with chronic alcohol consumption. ALDH2*2, a common dysfunctional polymorphism in the *ALDH2* gene, has been linked to an increased risk of cancer and heart disease. We tested the effect of ALDH2 deficiency on alcohol-induced AF in a murine model of chronic-binge ethanol feeding, with ALDH2*2 knock-in (KI) mice generated by a CRISPR/CAS9 system. In addition, right atrial appendages were obtained from eight patients with AF undergoing open heart surgery. The results showed that burst atrial pacing induced a greater susceptibility to AF in ALDH2*2 KI mice exposed to chronic ethanol intoxication than in wild-type mice, resulting from a higher degree of 4-HNE accumulation and collagen deposition in their atria. Alda-1 attenuated transforming growth factor beta 1 (TGF-β1) expression and collagen deposition in the atria and reduced AF inducibility. Patients with AF and the ALDH2*2 allele exhibited greater oxidative stress and substrate remodeling in their atria than non-carriers. In conclusion, ALDH2 deficiency may increase the risk of chronic alcohol and tachypacing-induced AF through the accumulation of 4-HNE and increased ROS production.

## 1. Introduction

Atrial fibrillation (AF) is the most common sustained cardiac arrhythmia and a major public health burden [1]. Accumulating evidence suggests a link between oxidative processes and AF [2], and many studies have demonstrated that the accumulation of cytotoxic and reactive aldehydes derived from reactive oxygen species (ROS) can also severely impair cardiac function [3,4,5]. Reactive aldehydes generated from lipid peroxidation of mitochondrial and plasma membranes, such as malondialdehyde (MDA) and 4-hydroxy-trans-2-nonenal (4-HNE), have been detected in almost all tissues that are under oxidative stress conditions. These aldehydes in turn form adducts with lipids, proteins and DNA, leading to their inactivation [6,7]. Mitochondrial aldehyde dehydrogenase 2 (ALDH2) is a key enzyme that metabolizes acetaldehyde (a toxic intermediate of ethanol metabolism) to acetic acid, and also detoxifies ROS-generated aldehyde adducts [3]. ALDH2 is expressed abundantly in the liver and lungs, and is also present in organs that require high mitochondrial content, such as the heart and brain [8]. Approximately 40% of East Asians carry a common ALDH2*2 polymorphism (known as rs671 or Glu487Lys), which has been shown to dramatically reduce enzymatic activity compared with ALDH2*1, a wild-type allele [9]. Carriers of the ALDH2*2 allele have characteristic acetaldehyde-induced facial flushing when drinking alcohol [10]. The E487K amino acid substitution at the dimer interface of tetrameric enzymes has been shown to result in disruption of co-enzyme NAD binding and reduce the catalytic activity of ALDH2*2 [11]. Individuals with the heterozygous ALDH2*1/*2 genotype retain only 10–45% of the enzymatic activity, and those with the homozygous ALDH2*2/*2 genotype have 1–5% of wild-type ALDH2 activity [12]. The ALDH2*2 allele has been linked to an increased risk of esophageal and other upper aerodigestive tract cancers among alcohol drinkers in Asians [13]. Higher incidence rates of insensitivity to nitroglycerin treatment for angina [14], coronary spasm [15], myocardial infarction [16,17], hypertension [18], stroke [19] and other oxidative-related neurodegenerative diseases [20] have also been associated with the ALDH2*2 polymorphism in previous studies. Previous meta-analyses have also consistently shown an increase in the risk of AF in individuals with chronic alcohol consumption [21], however, the mechanisms underlying this association are unclear. The aim of this study was, therefore, to assess the potential role of ALDH2 in protecting against AF. We hypothesized that ALDH2*2 allele–alcohol interactions may play a role in the development and self-perpetuation of AF by increasing ROS production and 4-HNE accumulation. A selective ALDH2 activator could therefore serve as an alternative treatment option for AF.

## 2. Results

### 2.1. Generation of ALDH2*2 Knock-In (KI) Mice

**Figure 1:**
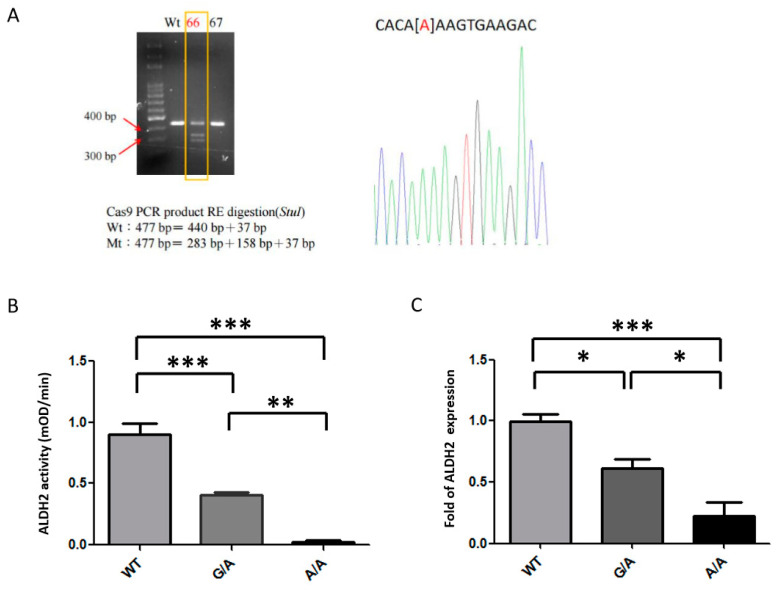
Genotyping and efficacy of Aldehyde dehydrogenase 2*2 knock-in (ALDH2*2 KI) mice. (**A**) Founder (lane marked by the box) mice containing the KI allele were confirmed by sub-cloning and direct sequencing analysis. [A], G-to-A substitution (**B**) Decreased ALDH2 enzymatic activities in the livers of the ALDH2*2 KI mice. (**C**) Decreased ALDH2 protein expression in the heart tissues of the ALDH2*2 KI mice. WT, wild-type; Mt, mutant; RE, restriction enzyme; * *p* < 0.05, ** *p* < 0.01, *** *p* < 0.001.

The ALDH2*2 KI mutation was generated using CRISP/CAS9 in mouse embryonic stem (ES) cells via the introduction of a G-to-A substitution in exon 12 of the endogenous *Aldh2* locus, producing a missense mutation orthologous to the human *ALDH2* (E487K) mutation. The KI allele was confirmed by PCR detection of the inserted remaining *StuI* site (Figure 1A) and by direct sequencing of genomic DNA (Figure 1B). ALDH2*2 KI mice generated from these ES cells were born at the expected Mendelian ratio, had a normal life span, and were healthy and fertile. The ALDH2*2 KI mice were confirmed to have decreased ALDH2 enzymatic activity in the liver (Figure 1B) and decreased ALDH2 protein expression in the heart (Figure 1C), consistent with previous studies indicating that ALDH2*2 KI mice had decreased ALDH2 expressions due to increased ALDH2 protein turnover in both human samples and murine samples with ALDH2*2 KI [22,23].

### 2.2. Increased 4-HNE Production in the ALDH2*2 KI Mice with Chronic Ethanol Intoxication

**Figure 2:**
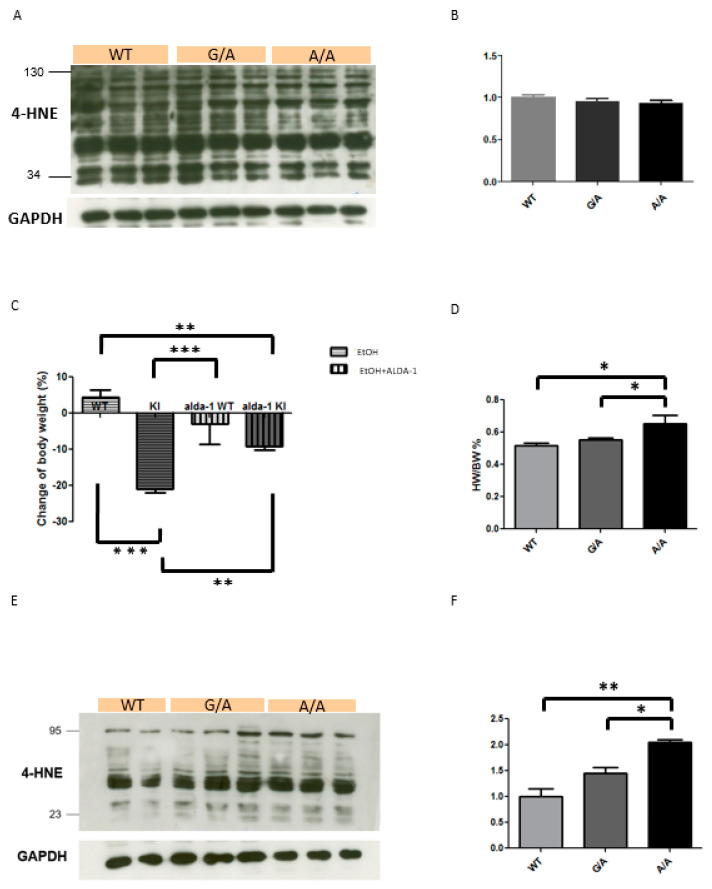
Chronic ethanol intoxication of wild-type (WT) and ALDH2*2 KI mice. (**A**) Representative Western blot and (**B**) quantification relative to GAPDH of 4-hydroxy-trans-2-nonenal (4-HNE) production in mice hearts with the indicated genotypes before chronic ethanol intoxication. (**C**) Body weight (BW) and (**D**) ratio of heart weight (HW) and BW of WT and ALDH2*2 KI mice treated with a liquid diet containing 5% ethanol with and without Alda-1 (20 mg/kg per day) for 10 days, then a single dose of ethanol (5 g/kg BW, 20% ethanol) at day 11. (**E**) Representative Western blot and (**F**) quantification relative to GAPDH of 4-HNE production in mice hearts with the indicated genotypes after chronic ethanol intoxication. Each value represents the mean ± SE of at least three independent experiments. KI, heterozygous and homozygous ALDH2*2 KI mice; EtOH, ethanol; * *p* < 0.05, ** *p* < 0.01, *** *p* < 0.001.

To study the chronic effects of ethanol consumption, we treated wild-type and ALDH2*2 KI mice with a liquid diet containing 5% ethanol for 10 days, and then a single dose of ethanol (5 g/kg body weight, 20% ethanol) at day 11. A previous study demonstrated that this chronic-binge ethanol feeding model is useful to study the early stage of alcoholic liver injury without an acute lethal effect and behavioral abnormalities [24]. Before chronic exposure to ethanol, heterozygous and homozygous ALDH2*2 KI mice exhibited similar 4-HNE production in cardiomyocytes compared with wild-type mice (Figure 2A,B). Although the ALDH2*2 KI mice exhibited a significant reduction in body weight (Figure 2C), there was a significant increase in the ratio of heart weight and body weight in the homozygous ALDH2*2 KI mice at day 11 after chronic ethanol treatment compared with the wild-type mice (Figure 2D). As shown in Figure 2, the ALDH2*2 KI mice had significantly higher 4-HNE accumulation in the heart when compared to the wild-type controls, indicating possible injury of cardiomyocytes caused by chronic ethanol treatment (Figure 2E,F).

### 2.3. Increased AF Inducibility in the ALDH2*2 KI Mice with Chronic Ethanol Intoxication

**Figure 3:**
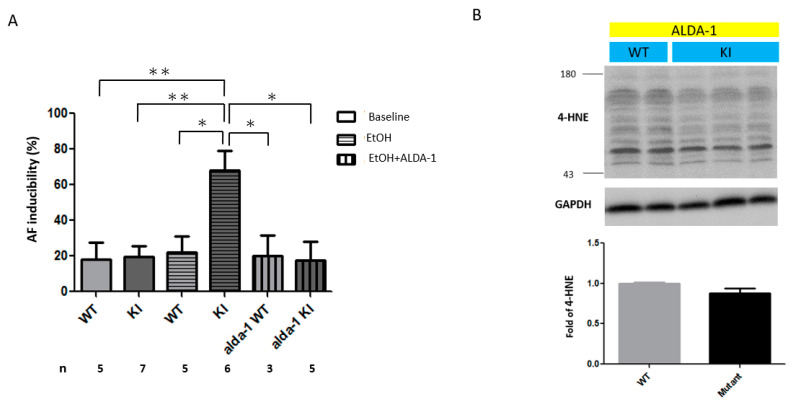
(**A**) Effect of transesophageal burst pacing on the occurrence of atrial fibrillation (AF) in wild-type and ALDH2*2 KI mice with chronic ethanol intoxication with and without Alda-1 treatment. (**B**) Effect of Alda-1 treatment on the production of 4-HNE in wild-type and ALDH2*2 KI mice with chronic ethanol intoxication. (**A**) The inducibility of AF was quantified by the ratio of pacing-induced AF episode in ten pacing bursts in each mouse (*n*, the number of mice in each experimental group). (**B**) Representative Western blot and quantification relative to GAPDH of 4-HNE production in mice hearts. Each value represents the mean ± SE of at least three independent experiments. WT, wild-type; KI, heterozygous and homozygous ALDH2*2 KI mice; EtOH, ethanol; * *p* < 0.05, ** *p* < 0.01.

We investigated the susceptibility to developing AF in the wild-type and ALDH2*2 KI mice using transesophageal burst pacing at the atrium before and after chronic ethanol consumption. Before chronic exposure to ethanol, heterozygous and homozygous ALDH2*2 KI mice exhibited a similar degree of AF inducibility compared with the wild-type mice. As shown in Figure 3A, the KI mice developed greater inducibility of AF than the wild-type controls after chronic ethanol treatment.

### 2.4. Increased Atrial Fibrosis in the ALDH2*2 KI Mice with Chronic Ethanol Consumption

**Figure 4:**
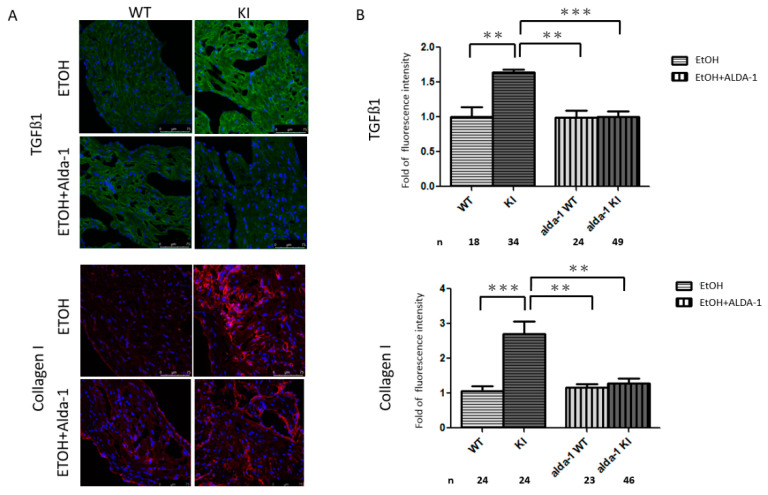
Atrial structural remodeling in wild-type and ALDH2*2 KI mice with chronic ethanol intoxication with and without Alda-1 treatment. (**A**) Representative confocal images showing the expression of transforming growth factor beta 1 (TGF-β1) and collagen I in the atria of mice. (**B**) Relative intensities of TGF-β1 and collagen I production were quantified. At least 10 random fields were chosen with scanning and averaging. Data are expressed as mean ± SE. *n*, the number of fields observed in each quantitative measurement; WT, wild-type; KI, heterozygous and homozygous ALDH2*2 KI mice; EtOH, ethanol, ** *p* < 0.01, *** *p* < 0.001.

Atrial fibrosis has been reported to greatly contribute to the pathogenesis of AF. In addition, acetaldehyde has been shown to be fibrogenic by upregulating the transcription of collagen I directly as well as indirectly by inducing the synthesis of transforming growth factor beta 1 (TGF-β1) in alcohol-induced hepatic fibrosis [25]. Therefore, we next investigated whether ALDH2 has a protective effect on collagen expression in the atria of mice after chronic ethanol consumption. We found that TGF-β1 expression and atrial fibrosis (indicated by increased collagen I generation) were more severe in the atria of the ALDH2*2 KI mice than in the wild-type controls after chronic ethanol consumption (Figure 4).

### 2.5. In Vivo Treatment with Alda-1 (ALDH2 Activator)

As shown in Figure 3A, the administration of the ALDH2 activator Alda-1 (20 mg/kg/day for 10 days) with ethanol significantly ameliorated the alcohol-related AF inducibility to transesophageal burst pacing in the ALDH2*2 KI mice. Alda-1 increased ALDH2 activity in the ALDH2*2 KI mice with chronic ethanol consumption (1.97 ± 0.15 vs. 1.28 ± 0.20 mOD/min, *p* = 0.039). In addition, Alda-1 mitigated the reduction in body weight caused by chronic ethanol consumption in the ALDH2*2 KI mice (Figure 2C). Moreover, Alda-1 reversed the 4-HNE accumulation (Figure 3B) and alleviated atrial remodeling by reducing the expression of TGF-β1 and fibrosis (Figure 4) after chronic ethanol consumption. Taken together, these findings suggested that ALDH2 may inhibit the expression of TGF-β1, collagen deposit and the consequent atrial structural remodeling in response to chronic ethanol consumption.

### 2.6. Association of the ALDH2*2 Polymorphism with Oxidative Stress and Structural Remodeling in Human AF Tissues

**Figure 5:**
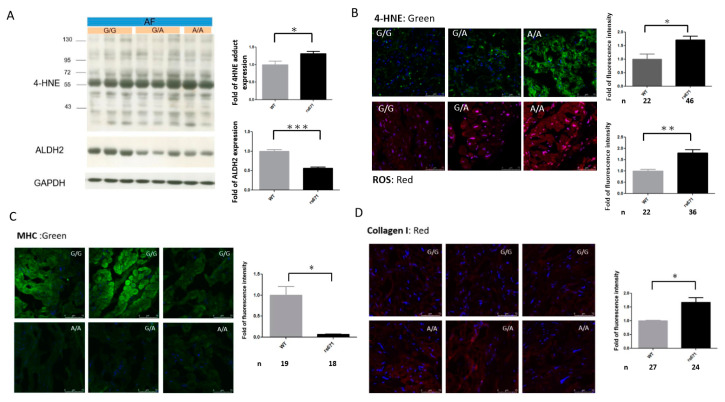
Associations between the ALDH2*2 polymorphism (rs671) and oxidative stress and atrial structural remodeling in human AF tissues. (**A**) Western blot and quantification relative to GAPDH of 4-HNE and ALDH2 expression in the homogenates of atria from AF patients with the indicated rs671 genotypes. Each value represents the mean ± SE of at least three independent experiments. Representative confocal images show 4-HNE (**B**), reactive oxygen species (ROS) (B), myosin heavy chain (MHC) (**C**) and collagen I (**D**) production in the atria of eight AF patients (3 wild-type (WT), 5 rs671 with 3 ALDH2*1*2 and 2 ALDH2*2*2). Relative fluorescence density was quantified (right). At least 10 random fields were chosen with scanning and averaging. N, the number of fields observed in each quantitative measurement; data are expressed as mean ± SE. * *p* < 0.05, ** *p* < 0.01, *** *p* < 0.001.

To determine whether ALDH2*2 affects AF-related oxidative stress and substrate remodeling, we further assessed the relationships between ALDH2*2 and oxidative stress and fibrosis in the atria of patients with AF. Consistently, we found that ALDH2 expression was significantly reduced (Figure 5A), and 4-HNE accumulation (Figure 5A,B) was significantly increased in the atria of AF patients with the ALDH2*2 allele compared to those with the wild-type. Moreover, oxidative stress (indicated by increased ROS generation, Figure 5B), myofibril degradation (indicated by decreased myosin heavy chain (MHC) expression) and collagen I deposit (Figure 5C,D) were more severe in the atria of the AF patients with the ALDH2*2 allele than in those with the wild-type. Taken together, these findings suggested that the ALDH2*2 polymorphism may affect the expression of ALDH2 and the consequent atrial structural remodeling responses in human AF tissues.

## 3. Discussion

In this study, we successfully generated a mouse strain with a mutation in murine *aldh2* corresponding to the E487K substitution in human *ALDH2* using a CRISPR/Cas9 method. Similar to previous mouse strains generated by homologous recombination [23,26], these mutants also recapitulated essentially all human phenotypes including impaired clearance of acetaldehyde, increased sensitivity to chronic alcohol-induced toxicity, and reduced ALDH2 expression due to a dominant-negative effect of the mutation. Our results showed that burst atrial pacing induced a greater susceptibility to AF in ALDH2*2 KI mice exposed to chronic alcohol intoxication than in wild-type mice. In addition, the ALDH2*2 KI mice with chronic alcohol intoxication exhibited a higher degree of 4-HNE accumulation in their hearts, as well as TGF-β1 expression and collagen deposition in their atria than the wild-type mice. Moreover, the effects of chronic ethanol consumption on 4-HNE accumulation, atrial TGF-β1 expression and fibrosis, as well as susceptibility to AF in the ALDH2*2 KI mice were reversed by an ALDH2-selective activator (Alda-1). We further demonstrated that AF patients with the ALDH2*2 allele exhibited greater oxidative stress and substrate remodeling in their atria than AF patients with the wild-type, which was reflected by increased ROS generation, myofibril degradation and collagen deposition in their atria. Our data suggest that ALDH2 may have a protective effect on AF-related remodeling and self-perpetuation.

A previous meta-analysis reported an 8% increase in the risk of AF for each 10 g/day increase in alcohol consumption or per drink and day [21]. In addition, a recent randomized and controlled trial showed a lower recurrence and burden of AF among patients who reduced their alcohol consumption than among matched controls at 6 months of follow-up [27]. However, the mechanisms underlying the association between alcohol consumption and the development and perpetuation of AF remain unclear. Studies in animals and humans have shown that acute and chronic alcohol intake leads to depression of cardiac function and may also result in conduction abnormalities and morphologic changes [28]. In addition, another study reported that acute moderate alcohol consumption in healthy men was associated with an increased interatrial electromechanical delay [29]. Long-term heavy alcohol consumption has been reported to lead to dilated cardiomyopathy with both supraventricular arrhythmias such as AF and also ventricular arrhythmias [30,31]. The association between alcohol consumption and AF may also be attributed to a shortening of the right atrial effective refractory period [32]. Other potential mechanisms include vagal activity [33] and increases in oxidative stress [34]. Our findings further suggest that ALDH2*2 allele–alcohol interactions may also play a role in the development of AF. In this study, the ALDH2*2 mutation reduced ALDH2 protein levels and caused enzyme deficiency in the atria, leading to inefficient detoxification of aldehydes and the accumulation of oxidative stress. We also showed that TGF-β1, an important mediator in AF-related atrial fibrosis [35], was upregulated in the atria of ALDH2*2 KI mice exposed to chronic alcohol intoxication. As a result, shortening of the atrial effective refractory period and atria fibrosis contributed to atrial remodeling and increased vulnerability to AF after chronic alcohol consumption.

A recent case–control study investigated the association between ALDH2*2 polymorphism and AF in Japanese patients undergoing AF ablation and demonstrated that the dysfunctional A allele of *ALDH2* was significantly negatively associated with AF [36]. This finding is contrary to expectations and reports of an association between the *ALDH2* dysfunctional A allele and cardiovascular diseases in East Asian populations [14,15,16,17,18,19]. The discrepancy could be because individuals with ALDH2*2 have lower levels of alcohol consumption and reduced likelihood of heavy drinking compared with individuals without ALDH2*2, which in turn lowers the risk of developing AF. In our study, the ALDH2*2 KI mice exhibited a greater body weight loss than the wild-type mice after chronic alcohol consumption. In other words, our findings suggest that alcohol abuse may predispose individuals with the ALDH2*2 allele to AF. In addition, the *ALDH*2 genetic variant rs2238152, which is in complete linkage disequilibrium with rs671 (ALDH2*2)*,* was found to be associated with progression to hypertension in a prospective Chinese cohort [37]. The risk associated with the rs2238152 T allele was strongest in heavy/moderate alcohol drinkers and was reduced in non-drinkers, indicating an interaction between *ALDH2* genetic variants and alcohol intake on the risk of hypertension, which is also an important risk factor for AF.

Tachycardia-induced oxidative stress has been proposed to be an important mediator in promoting and perpetuating AF [38]. Studies from our group and others have shown that oxidative stress mediates tachycardia-stimulated atrial remodeling [39,40,41]. Therefore, the responsiveness of an individual atrium to oxidative stress may determine its vulnerability to AF self-perpetuation. Our atrial sample findings in the AF patients further support the notion that ALDH2*2 leads to decreases in ALDH2 expression and activity, and that this may hinder the detoxification of reactive aldehydes in parallel with tachycardia, and subsequently increase oxidative stress and atrial remodeling. The human data in our study further support the animal findings, indicating that ALDH2 deficiency may lead to the accumulation of 4-HNE and consequently exacerbate AF-related oxidative stress in the atria.

There are several limitation to this study. First, we did not use non-invasive ECG Holter monitoring to evaluate whether a longer period of alcohol consumption was associated with a greater risk of developing incident AF in the ALDH2*2 KI mice. Second, the detailed molecular mechanism behind the ALDH2-related cardioprotection against alcohol-related AF was not elucidated. Further studies are needed to investigate whether elevated 4-HNE can cause the overexpression of matrix metalloproteinases through activation of the NF-kB pathway or promote oxidative stress-induced cardiomyocyte apoptosis or autophagy via either the MAPK or PI3K/Akt/mTOR signaling pathways in atrial tissue, as previously reported in other cardiovascular disease models [42,43,44].

## 4. Materials and Methods

### 4.1. Ethics Statement

The study protocols were approved by the Human Research Ethics Committee at Chang Gung Memorial Hospital (Chang Gung Medical Foundation Institutional Review Board 97-0756A3, 15 July 2008 and 100-3047A3, 1 December 2011) and were conducted in accordance with the Declaration of Helsinki Principles. Written informed consent was obtained from each subject. All animal experimental procedures were approved by the Institutional Animal Care and Use Committee of Chang Gung University (Taoyuan, Taiwan; IACUC No. CGU15-167, 28 March 2016), and the experiments were performed in accordance with the relevant guidelines.

### 4.2. Human Samples

Right atrial appendages were obtained from eight patients with AF undergoing open heart surgery. After excision, the atrial appendages were immediately frozen in liquid nitrogen and stored at −85 °C. Subsequently, genomic DNA from each subject was sent for genotyping for the rs671 polymorphism using TaqMan SNP Genotyping Assays obtained from Applied Biosystems (ABI, Foster City, CA, USA). The Supplemental Table S1 displays the baseline characteristics of these eight patients.

### 4.3. Generation of ALDH2*2 KI Mice Using CRISPR/CAS9

*ALDH2* is highly conserved in mice and humans. An ALDH2*2 KI mouse model on a C57BL/6J background with an inactivating point mutation in *Aldh2* was generated using a CRISPR/CAS9 system as previously described [45]. In brief, to introduce a single nucleotide substitution (G to A) within exon 12 of the *Aldh2* genomic fragment corresponding to the position of the human E487K mutation, three single guide RNAs (sgRNAs) were designed for a murine *Aldh2* target locus, and their efficiencies were tested in cell lines. One of them (5′-GGCGTACACAGAAGTGAAGACGG-3′) was chosen for the subsequent construction of RGEN (RNA-Guided Endonuclease) expression vector, which was used for the preparation of DNA templates for in vitro transcription of sgRNA. A T7 polymerase initiation site was added to the 5-prime end of the sgRNA. In order to prevent re-cut of the KI allele by RGEN and to create an *StuI* restriction site (underlined), Mouse-*aldh2*-ssDNA Donor encompassing the sgRNA recognition site (in *italics*) (5′-GAGTGGCAGGGAGCTGGGCGAGTATGGCCTGCA*GGCCTACACAAAAGTGAAGACCG*TACGTACACAGCCTTCAGACTCCGTGGCCTGGCCTCTC-3′) was custom synthesized (Life Technologies) to contain alternate codons that still maintained the translated protein sequence. Six-week-old C57BL/6J female mice were super-ovulated and mated with C57BL/6J males. Day 0.5 single cell embryos were isolated and given pronuclear micro-injections as previously described [46]. The embryos were co-injected with sgRNA at 50 ng/µL, recombinant Cas9 protein 100 ng/µL, and the Mouse-*Aldh2*-ssDNA Donor at 30 ng/µL in DNase/RNase free micro-injection buffer (1 mM Tris, 0.25 mM EDTA pH 7.4). Twenty to twenty-five injected embryos were transferred into the oviducts of day 0.5 pseudopregnant CByB6F1 recipient female mice. Genomic DNA was prepared from tail samples of founder mice and their offspring. To establish donor insertion, the mice were genotyped using PCR primers, (F:5′-AAAGGGAGAGGTGTGCTGTG-3′, R;5′-TCAGGCTGACCAACTCTGTG-3′), followed by *StuI* restriction. The resulting fragments were 440 and 37 bp for the wild-type, and 282, 158 and 37 bp for KI. The PCR products were then directly sequenced or cloned and sequenced.

### 4.4. Mice Maintenance

ALDH2*2 KI mice were bred and maintained in a barrier facility under pathogen-free conditions at Chang Gung University. Wild-type littermates of ALDH2*2 KI mice served as controls for all analyses. Mice were housed on a 12 h/12 h light/dark cycle and given food and water ad libitum. The mice were older than 8 weeks at the beginning of the experiments and were used in accordance with protocols approved by the Institutional Animal Care and Use Committee of Chang Gung University.

### 4.5. Ethanol Challenge

To study the chronic effects of ethanol consumption, 8- to 10-week-old wild-type and ALDH2*2 KI mice were fed a nutritionally adequate liquid control diet (Dyets, Inc.; Bethlehem, PA, USA) for 5 days, then divided into two groups: the ethanol group was fed a liquid diet containing 5% ethanol (Dyets, Inc.; Bethlehem, PA, USA) for 10 days, and the control group was pair-fed a control diet for 10 days. At day 11, the mice in the ethanol group were gavaged a single dose of ethanol (5 g/kg body weight, 20% ethanol), while mice in the control group were gavaged isocaloric dextrin maltose. All mice survived after chronic-binge ethanol feeding. The mice were euthanized 9 h post gavage when the serum levels of ALT and AST reached a peak according to Ki’s study [24]. The hearts and livers were collected and stored in liquid nitrogen for further experiments.

To evaluate whether the increased vulnerability to AF in the ALDH2*2 KI mice with chronic alcohol intoxication was affected by ALDH2, the ALDH2 activator, Alda-1, was given at a dose of 20 mg/kg per day to the mice with 10 days of ethanol intoxication.

### 4.6. ALDH2 Enzymatic Activity

The activity of ALDH2 was analyzed using a colorimetric Mitochondrial Aldehyde Dehydrogenase (ALDH2) Activity Assay Kit (ab115348; Abcam, Cambridge, UK) according to the manufacturer’s protocol using 200 μg of total protein from liver homogenate.

### 4.7. Western Blot Analysis

Equal amounts of proteins were extracted from tissue and subjected to sodium dodecyl sulfate-polyacrylamide gel electrophoresis. After transfer to nitrocellulose membranes (Perkin Elmer, Waltham, MA, USA), the proteins were incubated with primary antibodies against ALDH2 (ab108306; Abcam, Cambridge, UK), 4-HNE (ab46545; Abcam, Cambridge, UK), α-tubulin (sc-32293; Santa Cruz, Delaware Avenue, CA, USA), and GAPDH (sc-32233; Santa Cruz, Delaware Avenue, CA, USA). Signals were detected by electrochemiluminescence (sc-2048; Santa Cruz, Delaware Avenue, CA, USA) and quantified by densitometry. Data in the linear immunoreactive range were normalized to GAPDH or α-tubulin as a loading control.

### 4.8. Programmed Electrical Stimulation

Transesophageal stimulation was performed as described previously with some modifications [47,48]. After the mice had been anesthetized by injecting Zoletil (50mg/kg) and xylazine (10mg/kg) intraperitoneally, a 4F electrode catheter (ST. JUDE MEDICAL, MN, USA) was inserted into the esophagus and connected to an isolated stimulator (SI-200, iWork Systems Inc., WA, USA) for atrial pacing. The pacing programs and EKG recordings were managed using IX-TA-220 and LabScribe software (iWork Systems Inc., WA, USA). Amplitude of 1.5× diastolic capture threshold and a duration of 2 ms were used. A pre-test burst was performed with a cycle length of 100 ms for 10 s to ensure the capture of atrial stimulation, and then a pacing burst with a cycle frequency of 30 Hz for 3 s was repeated 10 times. After these series of pacing bursts, AF was defined as a period of rapid irregular atrial rhythm lasting for more than 3 s. AF inducibility was expressed as a ratio of pacing-triggered AF episodes/10 pacing bursts in each individual mouse.

### 4.9. Histology and Immunohistochemistry

Mouse and human atrial tissues were embedded in O.C.T compound (Sakura Finetek USA, Torrance, CA, USA). Five-micrometer cross-sections were prepared. Immunohistochemical and cytochemical analyses were performed by confocal microscopy using TGF-β1 (sc146; Santa Cruz, Delaware Avenue, CA, USA), collagen I (ab21286; Abcam, Cambridge, UK) and MHC (ab50967; Abcam, Cambridge, UK) primary antibodies followed by fluorescein isothiocyanate (FITC) or Cy3-conjugated secondary antibodies (Abcam, Cambridge, UK). Nuclei were visualized by DAPI staining. The expression levels of target proteins were calculated as protein-occupied area in the tissue divided by the nuclear area. For each analysis, at least ten random fields were chosen to observe. ROS in the atria were measured using the fluorescent dye, dihydroethidium, a cell-permeable indicator of ROS. The tissue samples were pre-incubated with 10 μmol/L dihydroethidium for 30 min at room temperature, and ROS-mediated fluorescence was observed under a confocal microscope (Leica TCS SP8, Wetzlar, Germany), with excitation at 543 nm using an argon laser and emission recorded using a long pass (>600 nm) filter set to acquire two-dimensional images (512 × 512 pixels).

### 4.10. Statistical Analysis

Continuous variables were expressed as mean ± SEM. Using the Kolmogorov–Smirnov test, all continuous variables were determined to be approximately normally distributed. Independent Student’s t test and one-way ANOVA with post hoc Tukey’s test were applied for two groups and multiple comparisons, respectively. A *p* value of < 0.05 using a two-sided test was considered to be statistically significant. All statistical analyses were performed using SPSS software, version 20.0 (SPSS Inc., Chicago, IL, USA).

## 5. Conclusions

In summary, we demonstrated that ALDH2 deficiency was associated with the susceptibility to tachypacing-induced AF in a murine chronic alcohol consumption model and AF-related oxidative stress and structural remodeling in humans. Because of the high prevalence of the ALDH2*2 allele among East Asian populations, East Asians may be more susceptible to the AF arrhythmogenic effect of chronic alcohol consumption. These findings provide further evidence supporting ALDH2 activation as another potential therapeutic target for the treatment of AF.

## Supplementary Materials

Supplementary materials can be found at https://www.mdpi.com/1422-0067/21/18/6678/s1.
