# Supplementary material for: Aldehyde Dehydrogenase 2 Ameliorates Chronic Alcohol Consumption-Induced Atrial Fibrillation through Detoxification of 4-HNE
Source: Int J Mol Sci. 2020 Sep 12;21(18):6678. doi: 10.3390/ijms21186678 (PMC7555032; doi:10.3390/ijms21186678)
Supplement: Supplementary file 1 [file ijms-21-06678-s001.pdf]

**Supplemental**

**Table 1. Clinical characteristics of patients with AF at the time of cardiac surgery**

| | | | | Underlying cardiac disease | | | | | | Previous medical history | | | | | | |
|---|---|---|---|---|---|---|---|---|---|---|---|---|---|---|---|---|
| **No.** | Age (yr) | Sex | rs671 genotype | Operative Indication | Duration of AF (yr) | DM | hypertension | LV Ejection Fraction (%) | LAD (mm) | Alcohol | β blocker | Digitalis | statins | Diuretics | ACE inhibitors or ARB | Calcium channel blockers |
| **1** | 47 | F | G/G | MS | 6 | - | - | 71 | 58 | - | + | - | - | + | + | - |
| **2** | 46 | F | G/G | MS | 2 | - | - | 75 | 51 | - | + | + | - | + | + | - |
| **3** | 47 | M | G/G | MS | 12 | - | - | 70 | 59 | - | - | + | - | + | - | - |
| **4** | 49 | M | G/A | AS+MS | <1 | - | - | 33 | 54 | - | - | - | - | + | - | - |

| | | | | | | | | | | | | | | | | |
|---|---|---|---|---|---|---|---|---|---|---|---|---|---|---|---|---|
| **5** | 56 | F | G/A | AS+MS | >1 | - | - | 73 | 56 | - | - | + | - | + | + | - |
| **6** | 56 | M | G/A | MR | <1 | - | - | 67.3 | 51 | + | + | + | - | + | - | - |
| **7** | 62 | F | A/A | MS | >4 | - | + | 70 | 50 | - | - | + | - | - | - | - |
| **8** | 49 | M | A/A | HOCM | 9 | - | - | 88 | 76 | - | + | - | - | - | - | + |

DM, diabetes mellitus; LV, left ventricle; LAD, LA diameter; ARB, angiotensin receptor blocker; ACE, angiotensin converting enzyme; AR, aortic regurgitation; AS, aortic stenosis; MR, mitral regurgitation; MS, mitral stenosis; HOCM, hypertrophy obstructive cardiomyopathy
